# Axillary lymphangioma that developed following COVID-19 vaccination: a case report

**DOI:** 10.1186/s40792-022-01488-5

**Published:** 2022-07-08

**Authors:** Soichiro Sasa, Hiroaki Inoue, Tomohiro Inui, Naoki Miyamoto, Mariko Aoyama, Kazumasa Okumura, Hiroaki Toba, Takahiro Yoshida, Miki Tezuka, Chieko Hirose, Yasuyo Saijo, Hisanori Uehara, Ayumi Izumori, Masako Takahashi, Mitsunori Sasa, Hiromitsu Takizawa

**Affiliations:** 1 Department of Thoracic, Endocrine Surgery and Oncology, Tokushima Graduate School of Biomedical Sciences, 3-18-15 Kuramoto-cho, Tokushima City, Tokushima 770-8503 Japan; 2 Division of Radiology, National Hospital Organization Higashi Tokushima Medical Center, 1-1 Oomukai-kita, Ootera-aza, Itano-cho, Itano-gun, Tokushima 779-0193 Japan; 3 Division of Pathology, Tokushima University Hospital, 3-18-15 Kuramoto-Cho, Tokushima City, Tokushima 770-8503 Japan; 4 Division of Surgery and Radiology, Tokushima Breast Care Clinic, 4-7-7 Nakashimada-cho, Tokushima City, Tokushima 770-0052 Japan

**Keywords:** COVID-19 vaccination, Lymphangioma, Axillary lymphadenopathy, Lymphadenitis, Breast cancer

## Abstract

**Background:**

Extensive vaccination programs are being implemented worldwide for coronavirus disease 2019 (COVID-19). With the spread of vaccination, swelling of the lymph nodes after vaccination is frequently seen. We encountered a patient who developed left axillary lymphadenoma following vaccine administration.

**Case presentation:**

The patient was a Japanese woman in her 80 s who had previously undergone surgery for right breast cancer. She received two injections of the Pfizer-BioNTech COVID-19 vaccine in her left arm. Approximately 3 months later, she complained of left axillary swelling, and imaging resulted in a diagnosis of left axillary lymphangioma. In accordance with the patient’s wishes, we performed axillary mass resection. The pathological diagnosis was lymphangioma.

**Conclusion:**

Our examination findings indicated that congestion of the axillary lymph vessels might have been caused by upper-arm injections of the COVID-19 vaccine.

## Background

Extensive vaccination programs are being implemented worldwide for coronavirus disease 2019 (COVID-19). According to the package insert for the Pfizer-BioNTech COVID-19 vaccine, the incidence of lymphadenitis as an adverse event is low [1]. However, unilateral axillary lymphadenopathy has been a common unsolicited adverse event after vaccination with either the Moderna or Pfizer-BioNTech COVID-19 vaccine [2]. Moreover, various organizations recommend that COVID-19 vaccination should be performed on the contralateral side of patients who have unilateral upper-arm edema or those who have undergone axillary surgery to prevent edema on the vaccinated side [3, 4]. Here, we report our experience with a Japanese patient who developed a left axillary lymphangioma following left upper-arm vaccination with a COVID-19 vaccine.

## Case presentation

**Fig. 1:**
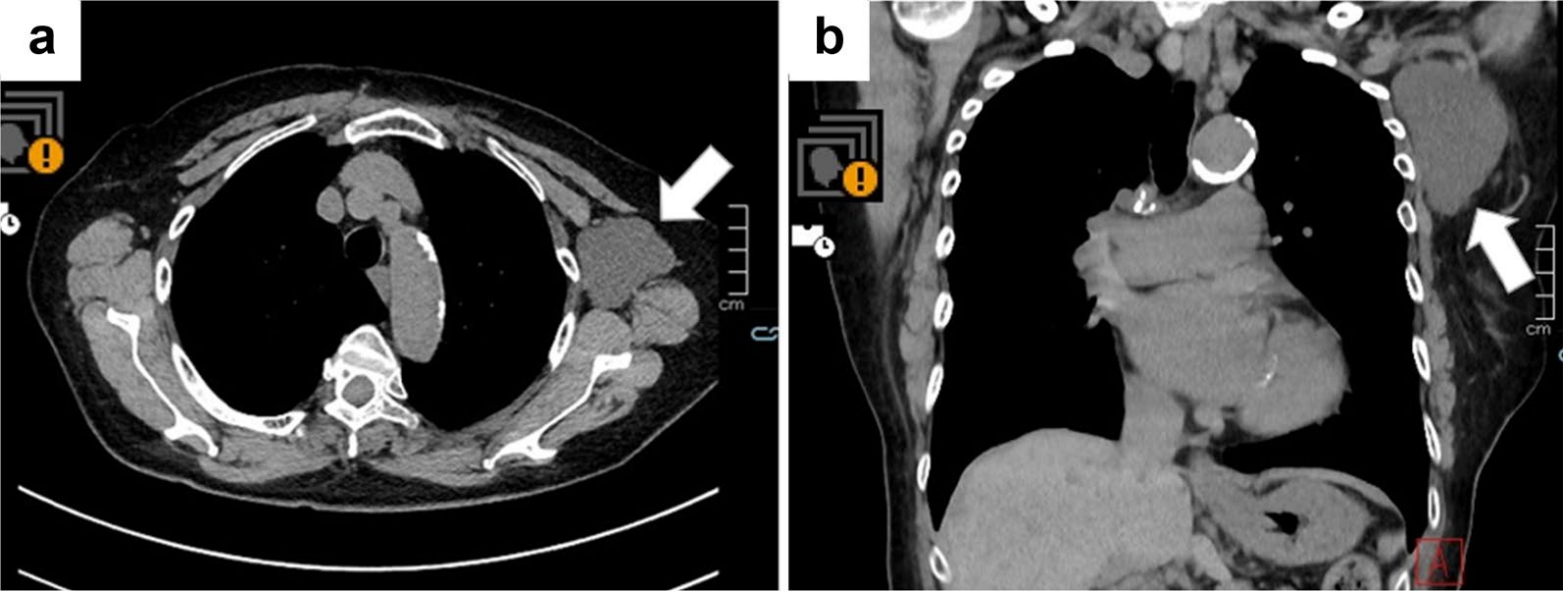
Non-contrast-enhanced computed tomography (NECT) findings (axial and coronal). Axial (**a**) and coronal (**b**) NECT scans showed a homogeneous, low-attenuation mass in the left axilla. The mass is slightly lobulated

A Japanese woman in her 80 s received a second injection of the Pfizer-BioNTech COVID-19 vaccine in her left deltoid muscle in 2021. She had a history of right breast cancer (T1N0M0) and had undergone breast-conserving surgery and sentinel node biopsy in her 70 s. Postoperative follow-up examinations were continued, and no sign of recurrence, including in the left axial region, was observed until 2021. There was no evidence of trauma to the left axial region. Her early adverse reaction following vaccination was mild pain at the inoculation site on the day of vaccination and the following day. However, 3 months after the second vaccination, she noticed left axillary swelling and visited the outpatient department at the Tokushima Breast Care Clinic. No lymphedema was found on her upper arm, but an elastic-soft left axillary mass was detected, and breast ultrasonography (US) revealed a 6-cm cystic mass. Computed tomography (CT) revealed a cystic mass without any solid pattern near the normal lymph nodes, and a lymphangioma was suspected (Fig. 1a, b).

**Fig. 2:**
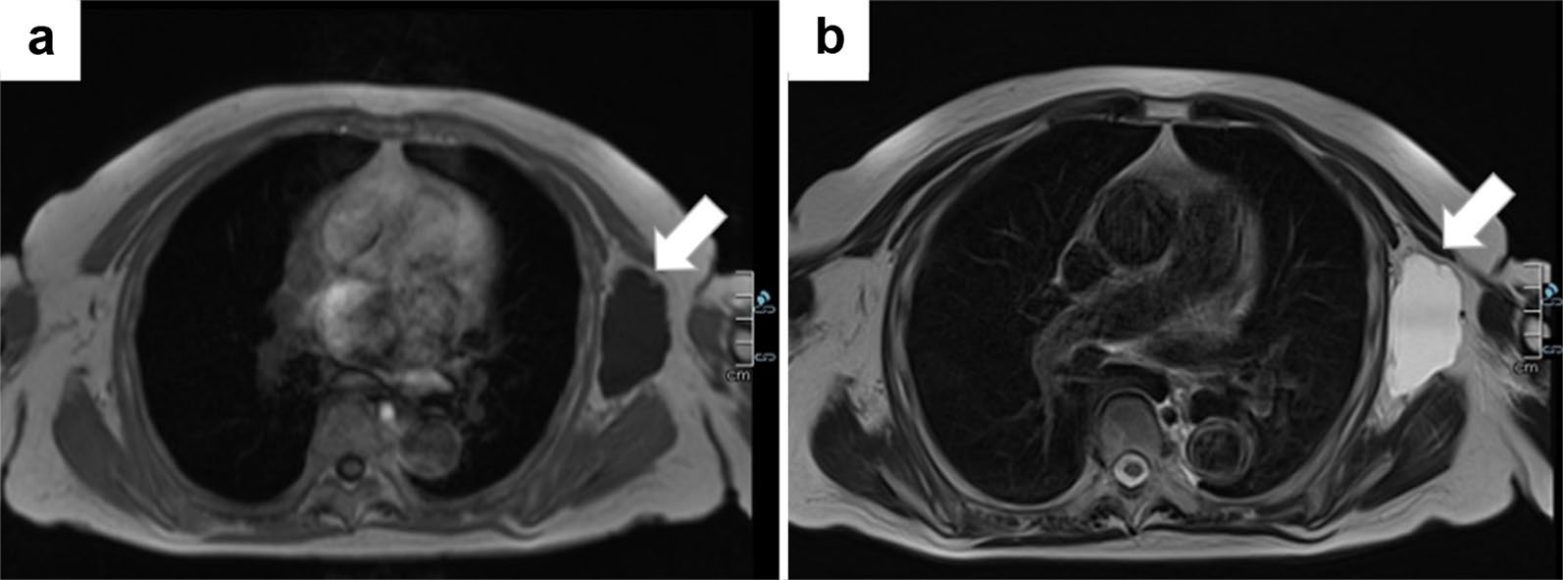
Magnetic resonance imaging (MRI) findings. Compared with the surrounding muscle, the left axillary mass showed a low signal on T1-weighted imaging (**a**) and high signal on T2-weighted imaging (**b**)

**Fig. 3:**
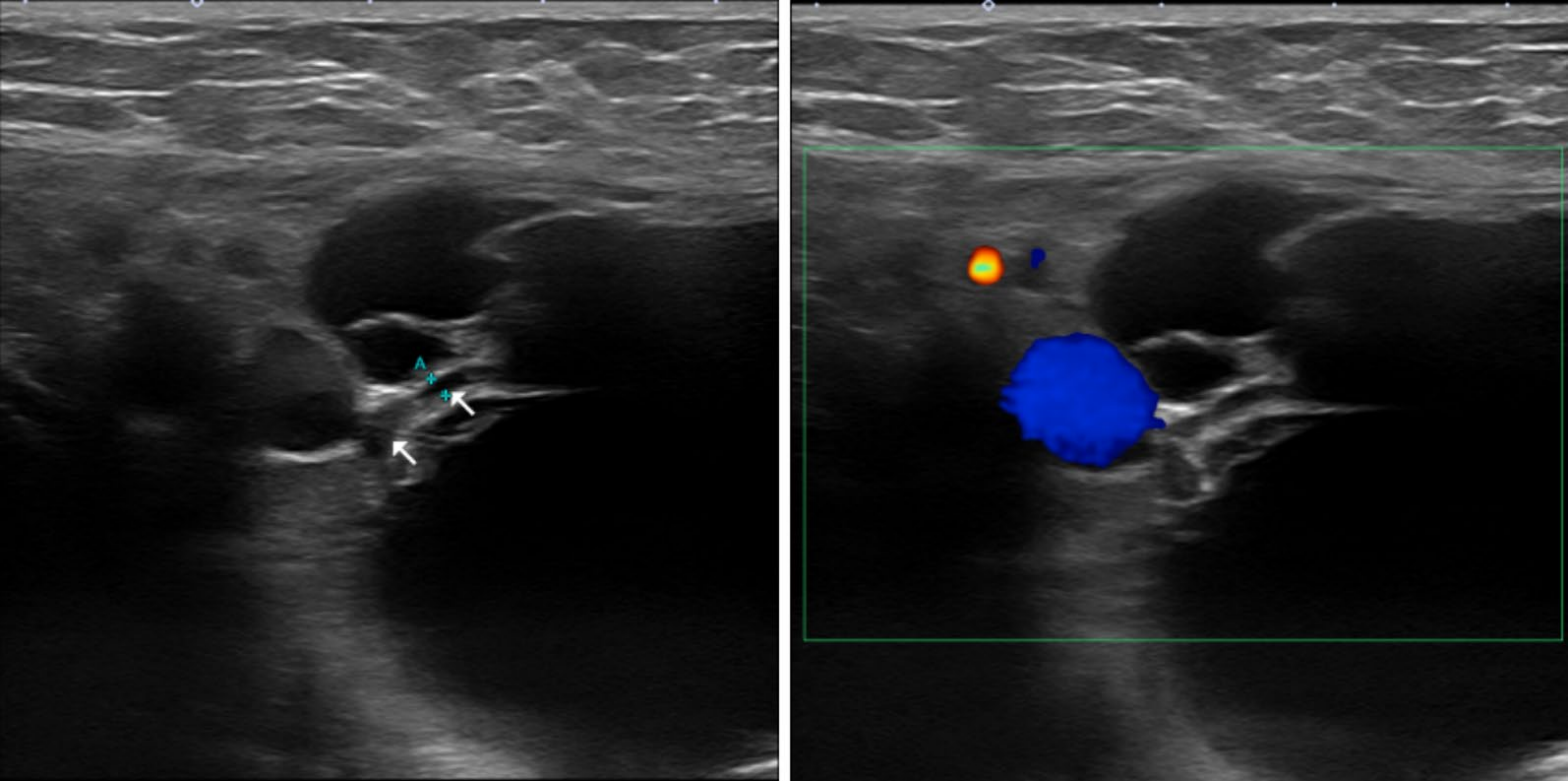
Breast ultrasonography findings. Breast ultrasonography confirmed a multilobulated cystic mass in the left axial region and branches (arrow) from the mass connected to the axillary vein

**Fig. 4:**
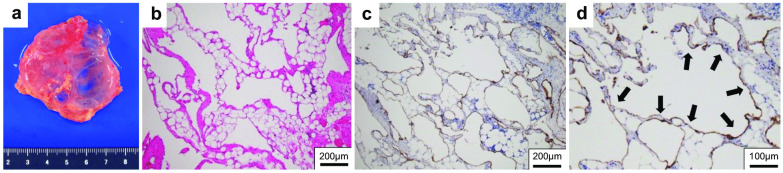
Pathological findings. Resected specimen of the axilla cystic tumor (**a**). In the cystic region, the inner surface was lined with flattened endothelial cells (**b**; hematoxylin–eosin staining). The endothelial cells were positive for D2-40 by immunostaining and determined to be lymphatic vessels (**c** and **d**)

Non-enhanced magnetic resonance imaging (MRI) revealed a 6.2 × 3.4 × 7.4-cm mass having a clear boundary, smooth surface and nearly homogeneous internal pattern. Compared with the surrounding muscle, the mass had a low signal on T1-weighted imaging (Fig. 2a) and a high signal on T2-weighted imaging (Fig. 2b). Internally, there was no solid pattern or high signal intensity on diffusion-weighted imaging, and a benign cystic mass (lymphangioma or synovial cystitis) was suggested. US revealed a multilobulated cystic mass and branches from the mass connected to the axillary vein (Fig. 3). Finally, a left axillary lymphangioma was diagnosed, and we suggested follow-up with drainage or resection. However, she expressed a strong desire for surgical resection because of the severe axillary discomfort. She underwent tumorectomy for the left axillary mass under general anesthesia. The mass did not adhere to the surrounding tissue and could be smoothly resected. On the cut surface, the tumor was a thin-walled transparent cyst filled with clear, yellowish fluid (Fig. 4a). Pathologic examination showed a large cystic region and anastomosing vasculature (Fig. 4b), both of which were lined with small, bland-appearing endothelial cells that were positive for D2-40 on immunostaining (Fig. 4c, d). Normal lymph nodes were found near the tumor. These findings were consistent with those of lymphangioma. The patient was discharged on the 7th day after surgery. The postoperative course is good as of the most recent follow-up, and the outpatient follow-up continues.

## Discussion

Lymphangiomas are tumorous lesions composed primarily of lymphatic cysts that occur mainly in children (most are congenital). The congenital cause is thought to be obstruction of the lymph vessels during the fetal period [5]. On the other hand, on rare occasions, lymphangiomas also occur in adults due to the obstruction of lymph vessels because of some cause (i.e., inflammation, trauma, radiotherapy, a thrombus) that leads to lymphatic fluid retention. The head and neck are the most common site for cystic lymphangiomas. Ultrasonographic findings include unilocular or multilocular anechoic mass. CT findings show non-enhancing cystic lesions with homogenous attenuation; visceral and osseous lesions often show contrast enhancement. Surgical resection may be indicated for large, deep lesions, especially if symptomatic [6, 7]. Here, we report a case of lymphangioma in the left axial region following injection of the Pfizer-BioNTech COVID-19 vaccine to the left upper arm.

We let the patient choose between conservative treatment and tumor removal. At the patient’s request, surgery was performed, which confirmed the diagnosis of lymphangioma. Although a causal relationship between vaccination and lymphangioma cannot be proven, we cannot rule out the possibility that the lymph vessels were congested because of an inflammatory response in the axillary lymph nodes and vessels. The following observations support this possibility: (1) the symptoms first manifested after the second vaccination; (2) there were no abnormal findings in the left axillary region in the 2008 CT examination or the 2021 US examination; (3) the left axillary region had incurred no trauma; and (4) no radiotherapy had been performed following the earlier surgery for the right breast cancer.

## Conclusions

Our findings indicated the possibility that occlusion of the axillary lymph vessels was caused by upper-arm injections of the COVID-19 vaccine. Further accumulation of cases is expected to elucidate the cause and pathogenesis of lymphangioma.

## Data Availability

The data are not available for public access because of patient privacy concerns.

## Abbreviations

COVID-19: Coronavirus disease 2019
US: Ultrasonography
CT: Computed tomography
MRI: Magnetic resonance imaging
